# Influence of different metal-organic frameworks on agronomic traits of faba bean plants

**DOI:** 10.1186/s12870-025-07478-7

**Published:** 2025-10-31

**Authors:** Noura E. Mahmoud, Reda M. Abdelhameed

**Affiliations:** 1 Genetic Resources Department, Biochemistry Unit, Desert Research Center, Cairo, Egypt; 2 Applied Organic Chemistry Department, Chemical Industries Research Institute, National Research Centre, Scopus affiliation ID 60014618, 33 EL Buhouth St., Dokki, Giza 12622 Egypt

**Keywords:** *Vicia faba*, Microelements, MOFs, Growth parameter, Stress indices

## Abstract

Micronutrient deficiencies in soil can significantly hinder plant growth and reduce crop production; therefore, foliar application of these micronutrients to plants becomes effective. Although metal-organic frameworks (MOFs) are frequently used in gas storage, adsorption, and catalysis, their application in agriculture has been rather uncommon. The purpose of this study was to ascertain how the growth parameters of faba bean (*Vicia faba* L.) plants were affected by nickel (Ni^2+^), chromium (Cr^3+^), cobalt (Co^2+^), and copper (Cu^2+^) MOFs. Using spectroscopic analysis, frameworks for Ni-BTC, Cr-BTC, Co-BTC, and Cu-BTC were described. These frameworks’ effects on the faba plants’ physiological stress indices, leaf chlorophyll and phenolic contents, fresh and dry weight of the roots and shoots, and shoot and root length were measured. According to our findings, Cu-BTC improved plant growth and development, resulting in a 37.79% increase in fresh shoot weight and a 40.42% increase in dry weight. Additionally, there was a 62.96% rise in the fresh weight of roots and a 75.0% increase in the dry weight of roots. The diameter of the stem was increased by 31.5% and the leaf surface area increased by 46.22%. There was an improvement in the amount of pigments, with carotenoids growing 1.91 times, chlorophyll a an increasing 1.59 times, and chlorophyll b increasing 2.07 times. On the other hand, Cr-BTC showed a negative impact on the growth of the plant; malondialdehyde (MDA) and H_2_O_2_ levels were raised by 4.54% and 14.21%, respectively, compared to the control. Although micronutrients are essential for plant growth and development, plants need them in small quantities. MOFs will deliver these micronutrients to plants in a controlled and efficient manner. MOFs can enhance nutrient uptake and reduce environmental impact by minimizing nutrient leaching and pollution.

**Supplementary Information:**

The online version contains supplementary material available at 10.1186/s12870-025-07478-7.

## Introduction

Mediterranean region and throughout Eurasia, including Egypt, Syria, Iraq, Iran, northern India, Pakistan, and southern China, faba bean is a staple food. Certain types of faba bean can be used for animal nutrition, and small-seeded beans are used for human nutrition in Egypt and other Arab nations. The analytical results on the fruit of faba bean indicate that it is rich in macro- and micronutrients. The macronutrients are 24% protein; the three protein fractions that were separated from the faba bean were albumin (7%), gluten (7%), and globulin (79%), also have a high lipid content, approximately 2% fat, and 50% carbohydrate, and around 700 calories per 100 g, varieties differ in their levels of tannin and fiber [1]. Furthermore, the micronutrients in faba bean grains, including polyphenols are abundant in faba bean [2]. Minerals including Ca, P, K, Mg, Na, S, Al, B, Ba, Co, Cr, Cu, Fe, Ga, Li, Mn, Ni, Pb, Sr, and Zn are also present in the fruits. Likewise, faba bean grains containing vitamins as folic acid, niacin, and vitamin C. As a result, fruits are a popular source of animal feed in Egypt and a protein-rich diet for humans [3]. Also, faba bean grains have free radical scavenging activity, making it a significant crop globally. In Europe, it is the second most popular food legume [2]. Higher meat costs and the desire for foods high in protein have caused individuals in less developed nations to switch to eating more grain legumes, particularly faba bean and green leafy vegetables that are richer in calcium and lower in phosphorus [4]. Because of their antioxidant and flavonoid content, beans have been used as a treatment to treat disorders of the kidneys, liver and ocular perceiver diseases [5].

Micronutrients are important for faba bean plant growth; micronutrients such as nickel (Ni) serve as ideal companions to strengthen the symbiotic relationship between soybeans and N_2_-fixing bacteria [6]. Therefore, a developing technique in modern agriculture is using nickel because it helps with nitrogen metabolism. Nickel can be applied in agriculture as soil fertilizer, foliar spray, or seed treatment. Recently, nickel was successfully applied to soybeans, it was found that soil, leaf, and seed treatments had positive impacts [7]. That application led to improvements ranging from 7 to 20% in biological nitrogen fixation, 1.5-fold in ureides, 14% in shoot dry weight, and yield increases of up to 1161 kg ha^−1^, photosynthesis increased 1.2 times, nitrogenase increased 1.4 times, and urease activity increased 3.9 times. Since nickel activates the urease enzyme, it is a necessary micronutrient for plants. Nickel contributes to the activation of a kind of glyoxalase I, which functions as a cofactor in the breakdown of methylglyoxal, a cytotoxic substance that is naturally generated by cellular metabolism [8]. By verifying that nickel is involved in the connection between the reduced glutathione cycle and reduced glutathione homeostasis, they further proposed that nickel might be important for plant antioxidant metabolism. The impact of spraying with varying nickel concentrations on the physiological and chemical properties of pot marigold was investigated [9]. They employed four distinct nickel nitrate concentrations: 0.07, 0.156, 0.234, and 0.312 mg L^−1^. The results demonstrated that varied amounts of nickel had substantial influence on the physiological and phytochemical features of marigold. The concentration of 0.234 mg L^−1^ was found to have the highest physiological benefits and to raise the total phenolic content, while the concentration of 0.156 mg L^−1^ was shown to be the most beneficial in increasing plant growth indices and antioxidant activity.

Micronutrients such as cobalt plays an important role in the process of fixing atmospheric nitrogen in many plants, especially those belonging to the legume family, which gives legumes their importance [10]. Cobalt is an essential part of vitamin B12, and cobalt is also an essential element for the work of many nitrogen-fixing enzymes. Cobalt increases agricultural yield, and cobalt is also a component of many proteins and enzymes and plays a role in plant metabolism. If there is a significant cobalt deficiency, plants may suffer from it. On the other hand, high levels of cobalt are harmful to plants. Increased levels of cobalt can impair photosynthesis and lead to iron deficiency in plants, which may lead to leaf drop. Foliar spraying of cobalt at varying concentrations of 5, 7.5, and 10 ppm on saline-grown faba beans was investigated [11]. In comparison to other cobalt rates, foliar spraying of cobalt at a concentration of 10 ppm produced the highest values of vegetative growth, yield, and their constituents. Comparing the 10 ppm cobalt spray to the control treatment, the results showed a 21.20% increase in chlorophyll a + b, a 9.65% increase in proline content, and a 6.86% increase in seed yield.

Micronutrients such as copper (Cu) is a crucial micronutrient for plant development under physiological settings. Cu is a structural component of regulatory proteins and is involved in oxidative metabolism, cell wall metabolism, hormone signaling, and the electron transport chain of respiration and photosynthesis [12]. For many crops, foliar spraying is a common method of applying different micronutrients. Because of its function in the cytochrome system and oxidative respiration, copper has been found to be necessary for nodulation [13]. In comparison to soybean and yellow lupin, faba beans provided the highest copper efficiency for the production of pods and grains [14]. Compared to wheat, chickpeas, and lentils, faba beans were more effective in using the copper in the local soil. Proteins with several Cu atoms in their structures include ascorbate oxidase, Zn/Cu superoxide dismutase, and Cu amino oxidase (8, 2, and 2 Cu atoms, respectively) [15]. Copper can move from old to new leaves and be absorbed as Cu^2+^ or Cu chelate, despite the fact that it is not extremely mobile in plants. Its concentration in plant dry mass is low, typically between 2 and 20 mg kg^−1^. However, the majority of plants are poisonous at concentrations in their dry mass that range from 20 to 100 mg kg^−1^ [16]. The chemical fruit characteristics of pomegranates, such as TSS, acidity, TSS/acid ratio, total reducing and non-reducing sugars, and vitamin C, were found to be positively impacted by copper foliar spraying at rates of 1, 1.5, 2, and 2.5%. Manfalouty pomegranates’ chemical fruit quality was greatly enhanced by copper spraying [17].

Metal-organic frameworks (MOFs) offer significant potential for sustainable agriculture due to their tunable pore structures and high surface area, enabling controlled release of agrochemicals, and enhanced nutrient delivery [18–21]. Copper-bound organic frameworks (Cu-MOFs) sprayed on oats cultivated in calcareous soils improved plant development by increasing biomass [22]. Total protein, water-soluble carbohydrates, free phenolic compounds in oat leaves, photosynthesis, and chlorophyll content were all improved by the spray treatment. Additionally, the Cu-MOFs spray treatment increased the oat grains’ contents of phenolic compounds, avenanthramides C, and vitamins E and K. The treatment also increased the output of grain and straw oats.

The availability of such micronutrients in soil was limited and caused various problems, including stunted growth, yellowing or spotting of leaves, poor flowering or fruit development, and reduced root growth; therefore, foliar spraying of these micronutrients was an ideal solution. Novel metal–organic frameworks (MOFs) enable controlled delivery of micronutrients to plants, which promotes early plant growth and enhances nutritional content. In this study, different MOFs based on micronutrients like Ni, Co, and Cu were sprayed on the faba bean plant, and their chemical, biochemical, and growth parameters were examined.

## Materials and methodology

### Materials

Metal chlorides (CrCl_3_·6H_2_O, 96%; NiCl_2_·6H_2_O, 99%; CoCl_2_.6H_2_O, 99%, 97%, CuCl_2_·6H_2_O, 99%), benzenetricarboxylic acid (BTC, C_9_H_6_O_6_, 98%), *N*,* N*-dimethylformamide (DMF, C_3_H_7_NO, 99%) and ethanol (C_2_H_5_OH, 99.9%) were purchased from Sigma–Aldrich.

### Preparation of MOFs

MOFs such as Ni-BTC, Cr-BTC, Co-BTC and Cu-BTC MOFs were synthesized hydrothermally under autogeneous pressure. The synthesis was carried out using a mixture of metal chlorides, benzenetricarboxylic acid and deionized water with a molar ratio of 1 MCl_3_: 0.5 BTC: 80 H_2_O (M = Ni, Cr, Co or Cu). The crystallization was carried out with microwave heating to take advantage of rapid syntheses of MOFs under microwave irradiation. After completion of the syntheses, the products were collected by cooling, centrifugation, washing with water and drying. To remove the BTC from the as-synthesized sample, purification was carried out at 70 °C using an ultrasonic generator in the presence of DMF. The purified product was collected after filtration and dried at 150 °C for 5 h.

### Characterization and instrumentation

The X-ray diffraction (XRD) patterns of the materials were examined using the (X’pert pro-Panalytical, Holland) operating system, with Cu Ka irradiation at 40 kV and 40 mA. With a range of 3° to 80° and a step width of 0.05°, the data were collected in the 2 θ. The surface morphology of Ni-BTC, Cr-BTC, Co-BTC and Cu-BTC MOFs were examined utilizing field emission scanning electron microscopy (FE-SEM QUANTA FEG250, Republic of Czech) at an accelerating voltage of approximately 20 KV. The elements were identified through the use of an energy dispersive x-ray (EDAX) study and a FE-SEM model (AMETA version). FTIR spectra were obtained using a Thermo Nicollet 5700 FT-IR spectrometer in the form of KBr pellets.

### Cultivation

Work being done in open area for experiments during the November 2023 season, the Desert Research Center (DRC) in Egypt carried out the experimental work in agricultural pots. The experiment’s goal was to evaluate the different effects of micro-elements fixed inside MOFs on the growth of faba beans. In this investigation, the varieties Sakha 4 and Giza 716 were employed. They came from the Agricultural Research Center’s Field Crops Institute in Giza, Egypt. In November 2023, the experiment on faba beans was conducted in pots in an open area with local weather conditions at the Desert Research Center (DRC), located in Cairo. The pot used in the experiment measured 25 cm in height and 30 cm in diameter. The soil type utilized was sandy soil, with 91.51% sand, 3.39% silt, and 5.10% clay [23]. Each pot included five faba bean seeds spaced 1.5 cm from the container’s surface. Irrigation was done with tap water. Twice a week, irrigation was carried out. And chemical analysis of the soil and water was performed; the soil pH was 7.62 the EC was 3.50 dS m^−1^, the water pH was 7.84, the EC was 0.0.43 dS m^−1^, and the mechanical dry sieving parameters were listed in the table (S1). A split-plot design was used for the experiment. Three duplicates of the cultivation were conducted.

### Foliar treatments

The experimental design was 5 types of groups for each variety of faba beans, group 1 was control, group 2 was Ni-BTC sprayed, group 3 was Cr-BTC sprayed, group 4 was Co-BTC sprayed, and group 5 was Cu-BTC sprayed. All pots were fertilized as follow 10 g/pot P, 10 g/pot N and 5 g/pot K. Sprinklers were used for the treatment of the plant with prepared materials. The operating pressures were 150 kPa, the height of the sprinkler riser was 0.5 m, and the nozzle diameter was 2.5 mm. The time of foliar application was in the morning between 7 and 9 am. Using ultra sonication, the powder was fully dissolved in 1000 milliliters of pure water. Treatment spraying was done for as follows: 0.5 ppm concentrations of Ni-BTC, Cr-BTC, Co-BTC, and Cu-BTC were sprayed foliar. Tween 20 was added as an improvement factor at a concentration of 0.05% after the culture process had been going on for 15 days. Seven days after the initial spray, a second spray was conducted. Vegetative samples were collected three, six, nine, twelve, and fifteen days after the second spray. The shoots and roots were separated at different ages, the fresh and dried weights were computed, the lengths of the shoots and roots were measured, the leaves were separated, their surface area was measured, and the diameter of the stem was measured. Together with the green sample obtained 15 days following the spraying procedure, a portion of the leaves was removed and dried in order to quantify the amount of total and free phenolics in the leaves. To determine the percent of antioxidant capacity, hydrogen peroxide, malondialdehyde, and chlorophyll, the fresh leaves were separated and kept in a deep freezer (−20 °C).

### Assay of growth parameter

Fresh weight for shoots and roots were estimated by its weighed used a laboratory balance. Samples were dried in an oven at 65 °C until stable, and then their dry weight was calculated by weighing the samples. Morphological traits were observed and characterized according to the National Test Guidelines [24].

### Determination of phenolic content

Hydrolysis and extraction protocol for the extraction of phenolic content was determined as follow, two equal portions of the homogenized powder (2 × 500 mg; ± 0.2 mg) were weighed and placed into plastic bottles featuring screw cups. To extract free phenolic compounds, aqueous methanol (1:1, v/v, 10 mL) was added to one bottle; to extract all phenolic compounds (total), an equal amount of acidified aqueous methanol (1:1, v/v, 88.8 g/L HCl, 10 mL) was added to the second bottle. After 15 min of sonication, the suspensions were incubated for 150 min at 82–83 °C, with 20 s of vortexing every 30 min. Following incubation, 10 mL of methanol was added to the cooled samples, and the mixture was vigorously stirred before the suspension was centrifuged for 10 min at 6000 rpm. The amount of free and total phenolic content was measured using supernatants. Determination of plant extracts’ phenolic content; The Phenolic content (free and total) were estimated using the folin-Ciocalteu method [25], 1mL of extract was mixed with 2.5 mL of 10% (w/v) folin-Ciocalteu reagent. After 5 min, 2 mL of Na_2_CO_3_ (75%) was subsequently added to the mixture and incubated at 50 °C for 10 min. afterwards, the sample was cooled and the absorbance was measured utilizing a UV Spectrophotometer at 765 nm against blank without extract. the outcome data were expressed as mg g^−1^ of gallic acid equivalents in milligram per gram of dry extract.

### Stress physiological indices

#### Determination of malondialdehyde (MDA)

The color of the adduct generated in the reaction between thiobarbituric acid (TBA) and malondialdehyde (MDA) in the TBA assay was used to quantify the quantity of lipid peroxidation in faba bean leaves. The supernatant was measured at 532 nm. The absorbance recorded at 532 nm was subtracted from each sample’s nonspecific absorption, which was measured at 600 nm. The concentration of the MDA-TBA adduct was calculated from the MDA standard curve and converted to µg g^−1^ fresh weight.

#### Determination of hydrogen peroxide (H_2_O_2_)

Hydrogen peroxide was determined based on potassium iodide oxidation by H_2_O_2_ in acidic media, this method was used to measure the hydrogen peroxide levels in fresh faba bean leaves. Spectrometric analysis was used to determine the color concentration. At 390 nm, the supernatant’s absorbance was determined. Using a standard curve, the hydrogen peroxide concentration was calculated and converted to µg g^−1^ fresh weight.

#### Antioxidant capacity assay

This is how 2,2-di-phenyl-1-picrylhydrazyl (DPPH) was measured: the methanolic extract of fresh faba bean leaves was combined with 2 mL of a 0.004% solution of DPPH in 80% methanol. About 2.5 mL of 80% methanol was used to homogenize about 0.5 g of fresh faba bean leaves. After cycling for 30 s, the reaction mixture was kept at room temperature for 30 min. Using spectrophotometry, the absorption wavelength of the mixture was determined to be 517 nm. The following equation was used to determine how well a substance could scavenge radicals:

$$\mathbf{RSC}\boldsymbol{\%} =\boldsymbol{[(}\mathbf{A}_{\mathbf{blank}} - \mathbf{A}_{\mathbf{sample}}\boldsymbol{)}\ / \ \mathbf{A}_{\mathbf{blank}} \boldsymbol{]} \times \boldsymbol{100}$$

where (RSC%) = percent of radical scavenging capacity, A_blank_ is the absorbance of methanolic DPPH solution, and A_sample_ is the absorbance of plant methanolic extract.

### Statistical analysis

The data was subjected to two-way ANOVA and the differences between means at the 5% probability level were determined using Duncan^’^s new multiple range test. The software SPSS, version 16 (SPSS, Richmond, USA) was used [26].

## Results and discussion

### Characterization of the MOFs

**Fig. 1:**
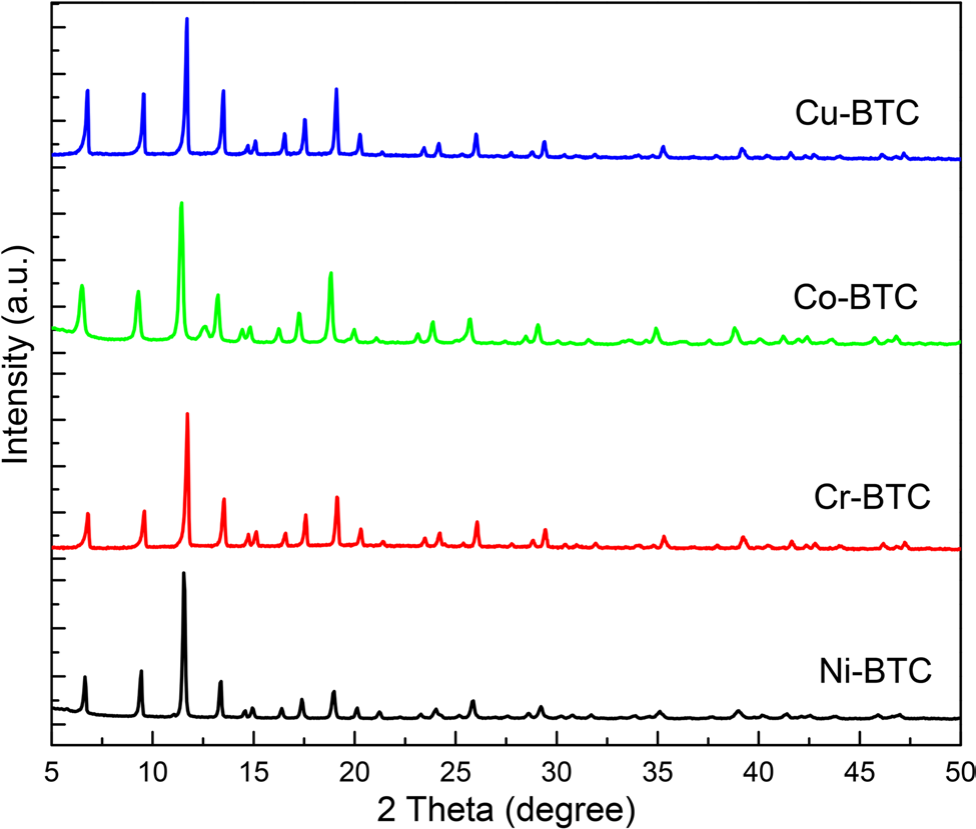
PXRD of the prepared MOFs Ni-BTC, Cr-BTC, Co-BTC and Cu-BTC

Figure 1 displays the Ni-BTC, Cr-BTC, Co-BTC and Cu-BTC MOFs experimental powder X-ray diffraction (PXRD) patterns. As can be observed, the phase purity of the as-synthesised samples is confirmed by the strong agreement between the previous work and experimental PXRD results [27]. The Ni-BTC, Cr-BTC, Co-BTC and Cu-BTC MOFs showed no significant changes in the crystal phase. This suggests that the compounds have the same structure with high crystalline phase and purity.

**Fig. 2:**
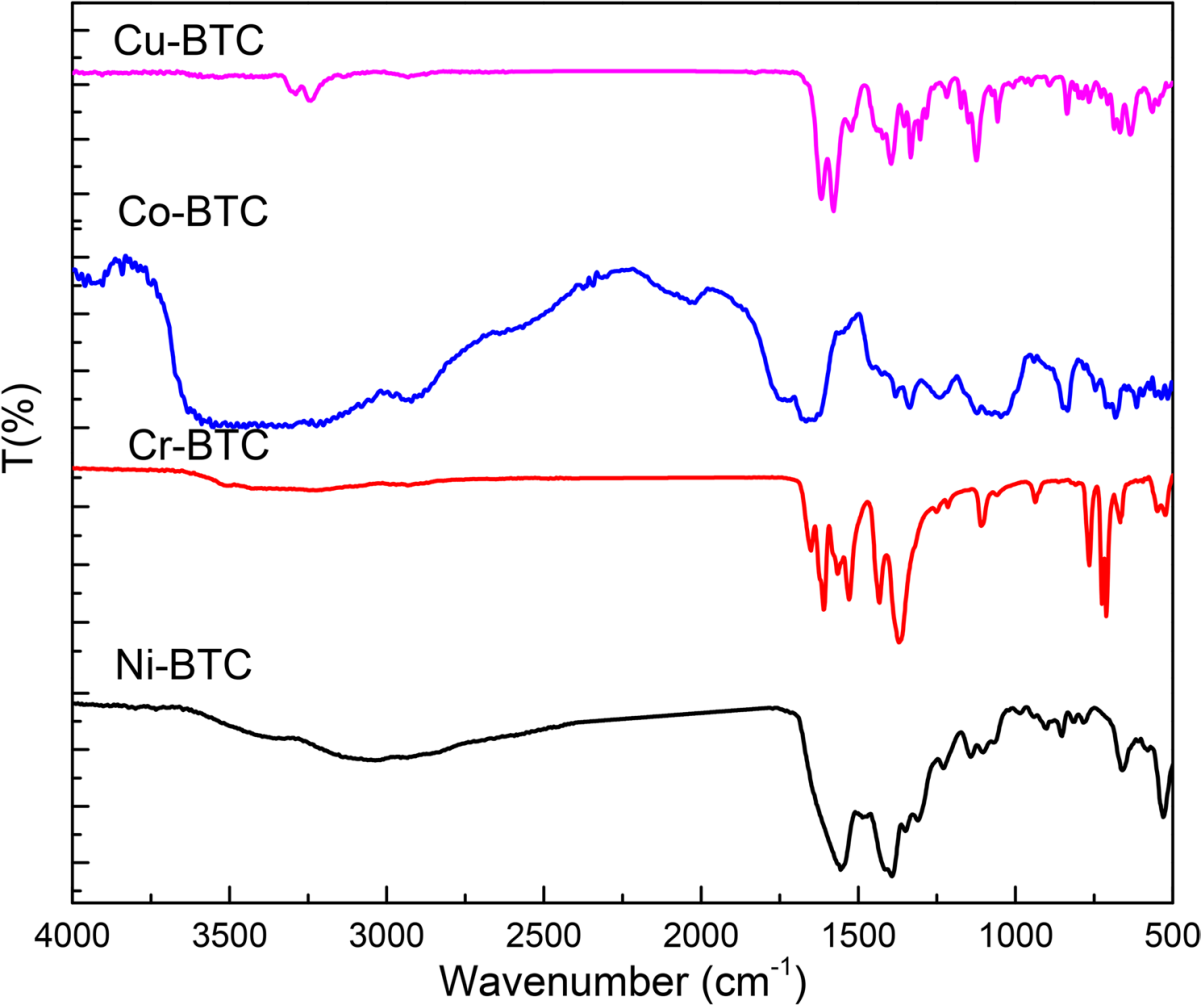
FT-IR of Ni-BTC, Cr-BTC, Co-BTC and Cu-BTC MOFs

The FT-IR spectra obtained for prepared materials are shown in Fig. 2. At 1800–1680 cm^−1^, these bands is relatively due to the adsorption bands of carboxylate group [28]. The benzene ring of BTC linker appeared at 1645–1550 cm^−1^ (asymmetric vibration) and 1470–1390 cm^−1^ (symmetric vibration) [29]. Additionally, the bands at 1100 cm^−1^ and in the area of 866–715 cm^−1^ comprise stretching vibrations of C-C groups as well as in-plane and out-of-plane deformation vibrations of the C-H groups in the benzene ring [29].

**Fig. 3:**
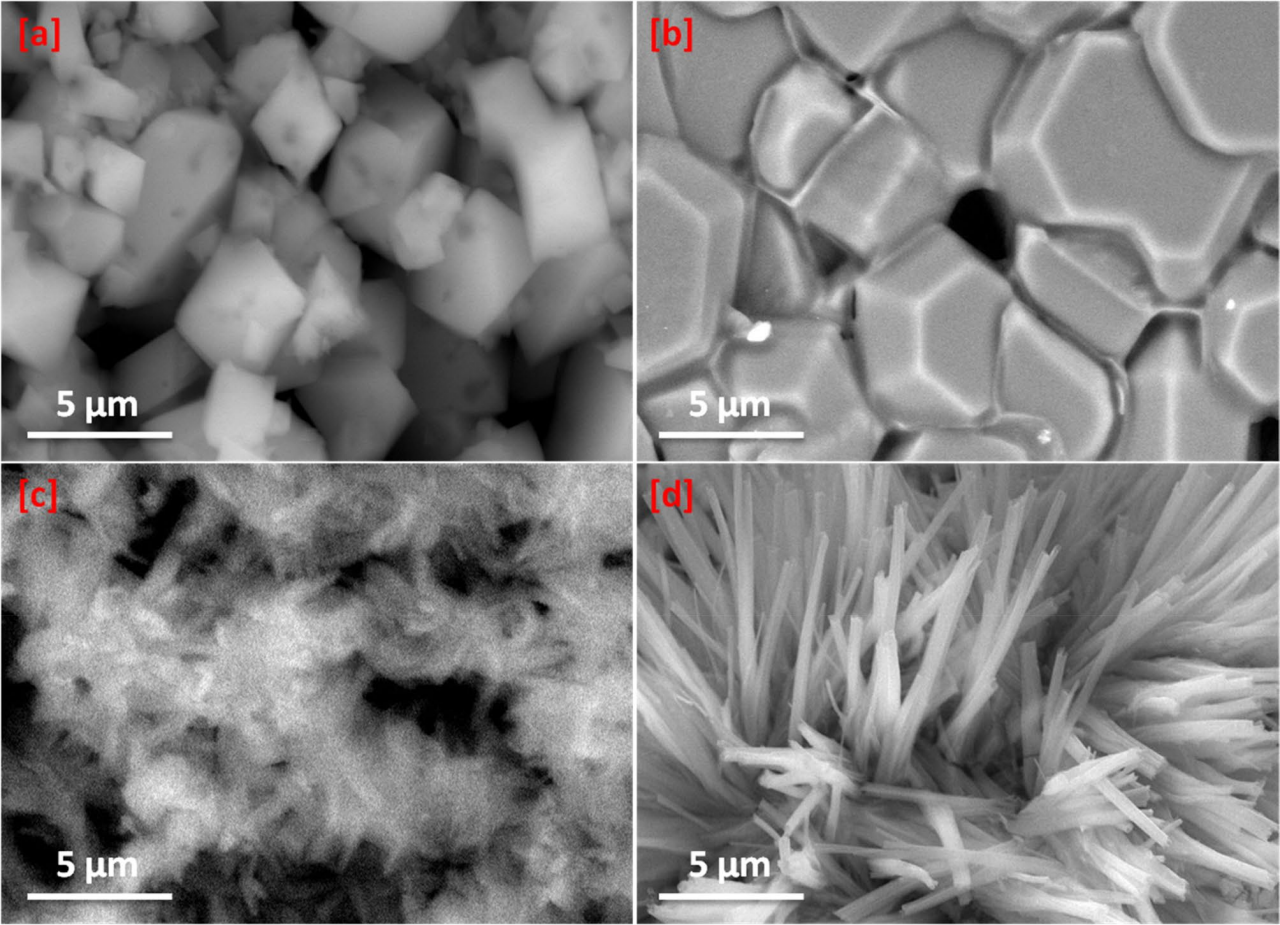
SEM images of the prepared MOFs (**a**) Cu-BTC, (**b**) Co-BTC, (**c**) Ni-BTC and (**d)** Cr-BTC

**Fig. 4:**
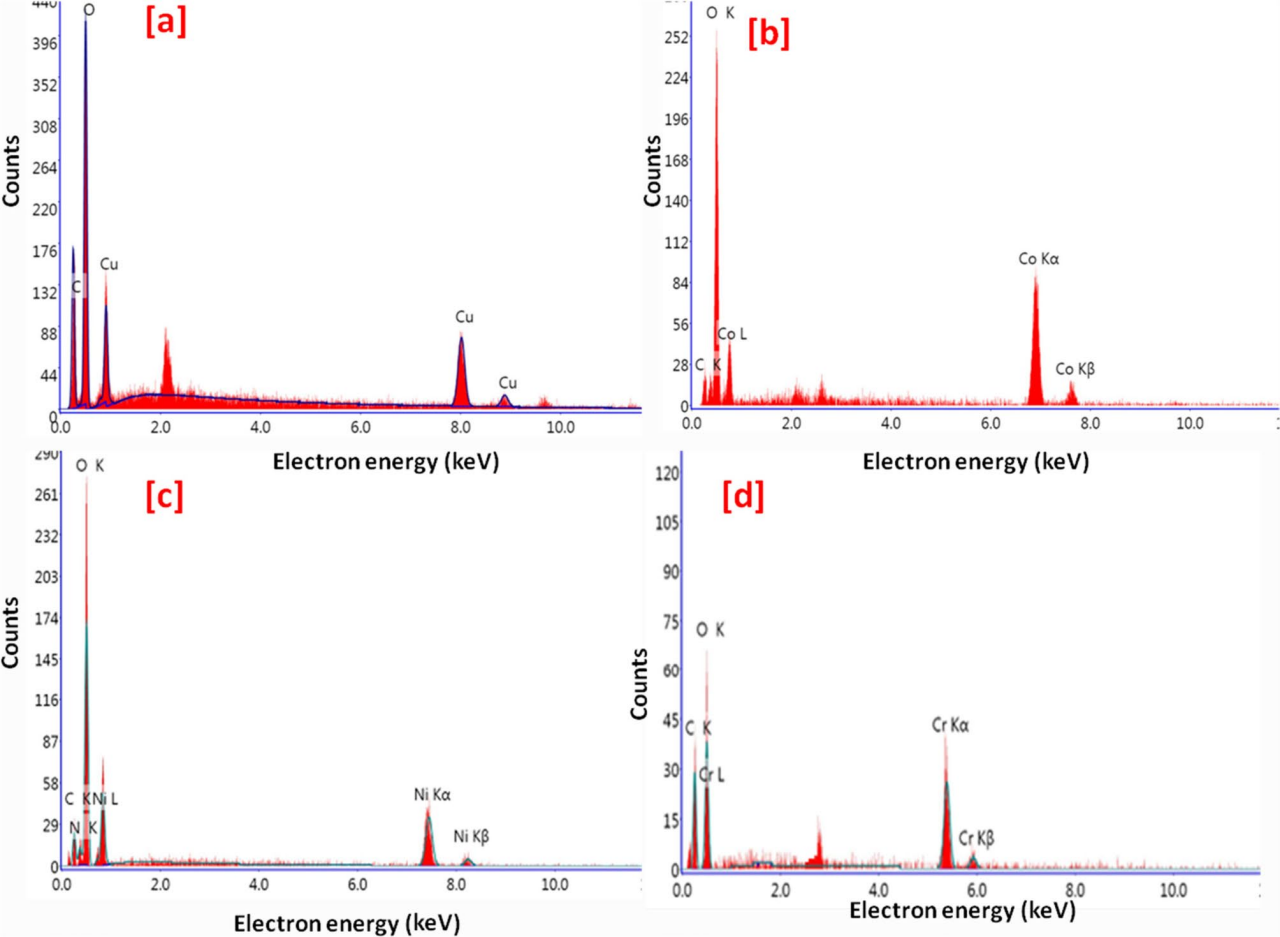
EDX images of the prepared MOFs (**a**) Cu-BTC, (**b**) Co-BTC, (**c**) Ni-BTC and (**d**) Cr-BTC

The morphology studies of Ni-BTC, Cr-BTC, Co-BTC and Cu-BTC MOFs were displayed in Fig. 3. Cu-BTC showed pyramidal shape (Fig. 3a) with good uniformity with a diameter of approximately 100 nm. Figure 4a showed the EDX spectrum of Cu-BTC, it showed the content of copper, oxygen and carbon atoms. Co-BTC morphology showed cubic particles with size of 1–10 μm (Fig. 3b) and the EDX spectrum of Co-BTC sample showed the presence of cobalt, oxygen and carbon atom (Fig. 4b). As shown in Fig. 3c, Ni-BTC showed regular fiber shaped micromorphologies. The EDX spectrum of Ni-BTC (Fig. 4c) showed Ni, O and C atoms. The morphology of Cr-BTC showed needle shape (Fig. 3d), and EDX spectrum in Fig. 4d showed the presence of chromium in addition to carbon and oxygen atoms.

### Growth parameters

**Fig. 5:**
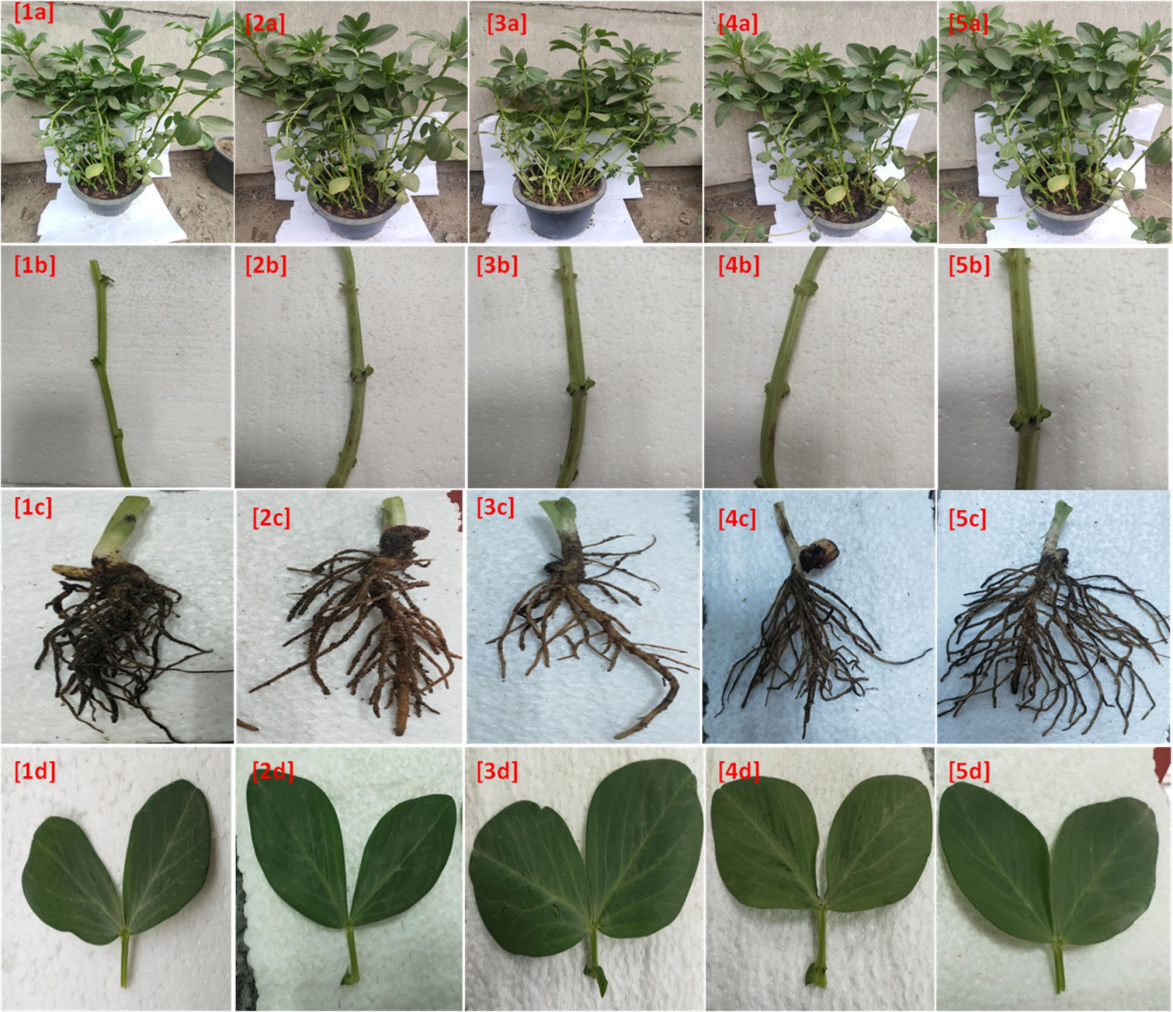
Optical images of the vegetative growth of faba bean (**a**) whole plant, (**b)** stem diameter, (**c**) root system shape and (**d**) leaf size after 15 days of external spray treatments: [1] control [2], Ni-BTC [3], Cr-BTC [4], Co-BTC and [5] Cu-BTC

The vegetative growth of beans following external spraying with Ni-BTC, Cr-BTC, Co-BTC, and Cu-BTC is depicted in Fig. 5. When compared to plant development without any external spraying, Fig. 5a illustrates a gradient in the effectiveness and quality of vegetative growth of entire faba bean plants. Cu-BTC spraying produced higher plant growth, followed by Co-BTC, Ni-BTC, and Cr-BTC sprays. The stem diameter, root system shape, and leaf size are displayed in Fig. 5b and c, and 5d, respectively. Every figure demonstrated that the spray treatment including Cu-BTC and Co-BTC was the most effective in enhancing the external look of the root and vegetative systems, followed by Ni-BTC and then Cr-BTC.

Compared to untreated plants of the same age, the greatest enhancement in shoot lengths occurred 15 days post-application of Cu-BTC treatments, succeeded by Co-BTC and Ni-BTC, with increases of 18.51%, 16.26%, and 15.92%, respectively, as illustrated in Table 1. The comparison of the two varieties indicated that shoot length for both faba bean varieties increased with age. Furthermore, in terms of shoot length, the Giza 716 variety performed better than the Sakha 4 variety. Regarding the interplay between varieties and spray treatments, the table’s data show that, following three, and twelve days of spraying, the Cu-BTC treatment outperformed with the Giza 716 variety. Furthermore, the Co-BTC spray treatment outperformed the other treatments with the Giza 716 variety at ages 6 and 9 days after exogenous applications, while the Cr-BTC spray treatment outperformed the other treatments at age 15 days after external spraying. The findings of this study are entirely in line with those of earlier research [10, 11, 30]. Numerous morphological, physiological, and biochemical processes in plants depend on Cu, an essential element. Plant growth rate is accelerated by copper, which is essential for signal modulation, protein transport, and iron and lipid metabolism [31]. As a cofactor in the active sites of numerous enzymes and in the electron transfer involved in hormone signaling, copper also aids in the growth and development of plants [32]. Cobalt’s significance in lengthening the vegetative group could also result from its significant biological role as a component of vitamin B12 and its derivatives. For numerous enzymes, cobalt serves as a cofactor. The synthesis of certain proteins, which promote plant growth, depends critically on cobalt [10, 33, 34]. Cobalamin, often known as vitamin B12, is necessary for rhizobia and other nitrogen-fixing bacteria to fix atmospheric nitrogen and supply plants with nitrogen macronutrients. Iron, nickel, and zinc interact with cobalamin to maintain cellular equilibrium. Low amounts of cobalt are necessary for plant growth, particularly for leguminous crops. When plants are cultivated in a medium with a significant drop in cobalt, plants exhibit choline shortage, and a rise in the element is harmful to the plant [10].

**Table 1:** Effect of foliar application of MOFs on shoot length dynamics in faba bean plants over time

Foliar applications	Shoot lengths (cm)														
	Three days			Six days			Nine days			Twelve days			Fifteen days		
	Sakha 4	Giza 716	Mean	Sakha 4	Giza 716	Mean	Sakha 4	Giza 716	Mean	Sakha 4	Giza 716	Mean	Sakha 4	Giza 716	Mean
Control	26.43 e	22.67 f	24.55 D	33.53 d	32.33 d	32.93 D	35.13 g	34.90 g	35.02 D	36.60 f	46.13 c	41.37 D	40.17 f	47.87 d	44.02 D
Ni-BTC	31.17 d	35.63 b	33.40 C	37.10 c	42.70 b	39.90 B	40.50 e	45.13 b	42.82 B	41.90 e	51.53 a	46.72 B	47.53 d	54.53 b	51.03 B
Cr-BTC	33.13 c	30.30 d	31.72 C	33.90 d	41.73 b	37.82 C	36.53 f	46.80 b	41.67 C	40.27 e	51.07 b	45.67 C	43.73 e	56.57 a	50.15 C
Co-BTC	33.70 c	37.57 a	35.63 B	35.00 c	46.57 a	40.78 B	36.77 f	50.30 a	43.53 A	44.13 d	49.30 b	46.72 B	47.77 d	54.60 b	51.18 B
Cu-BTC	35.40 b	40.47 a	37.93 A	41.47 b	44.50 a	42.98 A	42.73 d	44.73 c	43.73 A	44.40 d	52.80 a	48.60 A	49.77 c	54.57 b	52.17 A
Mean	31.97 A	33.33 A		36.20 B	41.57 A		38.33 B	44.37 A		41.46 B	50.17 A		45.79 B	53.63 A	

Values followed by the same letter in columns are not different at *p* < 0.05 by Duncan’s multiple range tests

Following sample collection at various intervals during the external spraying process 3, 6, 9, 12, and 15 days Table 2 illustrate the effect of spraying treatments on the length of the root system. The data showed that when the age stage increased for both faba bean varieties, the length of the root system increased as well. The findings also showed that the spray treatments containing Cu-BTC, and Co-BTC were the most successful in lengthening the root system across the different age stages when compared to the control. For each age stage independently, samples obtained after 3, 6, 9, and 15 days of Cu-BTC spraying demonstrated an improvement in root lengths that was 1.31, 1.33, 1.31, and 1.36 times larger than the control group, respectively. Regarding the comparison between the two varieties, it was observed that there were no discernible differences between them at any of the several maturity stages except, at 6 and 15 days following spraying, it was found that the Sakha 4 outperformed better than the Giza 716 variety in terms of root system length. Referring to the interaction between spray treatments and varieties, after three days of spraying, it was observed that the maximum mean value of root length of the Sakha 4 variety increased with Co-BTC and Cu-BTC spray treatments. The Sakha 4 variety also outperformed with the Co-BTC spray treatment in terms of improving the average root length at age stages 9, 12, and 15 days after spraying. In the same direction, after 6, 9 and 15 days of spraying, the Giza 716 variety performed better than the other variety with the Cu-BTC spray treatment in terms of growing root length. Also, the Giza 716 variety showed the greatest increase in root length following 3 days with Cr-BTC spray treatment when compared to untreated plants. The findings good match with those of earlier research [35, 36], they found that by regulating hormones in plant root cells, such as melatonin, auxin, and abscisic acid, Cu ions have been shown to modify the rate of root meristem cell proliferation, which in turn affects root development. Copper is necessary for node formation [37]. Additionally, root growth is also improved when cobalt is present in the soil in trace amounts [10]. A number of enzymes that may be crucial to the microbial activity surrounding the rhizosphere that promotes root growth are formed by cobalt [38, 39]. On the opposite side, the findings might not match those of earlier research [40, 41], which found that chromium inhibits plant root system growth. Cr may impede root growth by inhibiting cell division and reducing cell size in the long area. The findings of this study was matched with previous work that verified the chromium, when included in the culture medium at a concentration of 5 mg L^−1^, enhances the root growth of *Medicago sativa* seedlings [42]. *Convolvulus arvensis* seedlings’ root length increased when 0.238 mg/L of chromium and nitrogen were added simultaneously [43]. According to recent research in multiomics, chromium is essential for plants. Cr increases the synthesis of ROS and Ca^2+^, which in turn activates calcium-dependent protein kinase and NADPH oxidase, both of which are critical for later signaling cascades. Additionally, Cr is crucial for the stimulation of several genes involved in defense against metal stress, such as AP2/ERF TF and WRKY. Furthermore, phosphate kinase genes such PP2C A, PP2C-D, and PP2C-F are stimulated by Cr [44].

**Table 2:** Effect of foliar application of MOFs on root length dynamics in faba bean plants over time

Foliar applications	Root lengths (cm)														
	Three days			Six days			Nine days			Twelve days			Fifteen days		
	Sakha 4	Giza 716	Mean	Sakha 4	Giza 716	Mean	Sakha 4	Giza 716	Mean	Sakha 4	Giza 716	Mean	Sakha 4	Giza 716	Mean
Control	6.31 e	7.57 d	6.94 D	7.89 f	8.36 e	8.13 D	8.59 g	9.25 f	8.92 D	9.14 g	9.83 f	9.48 D	10.03 e	10.80 e	10.41 D
Ni-BTC	8.83 b	8.83 b	8.83 B	10.93 a	9.43 d	10.18 B	11.66 b	10.67 c	11.16 B	12.59 a	11.23 d	11.91 B	14.74 b	12.55 c	13.65 B
Cr-BTC	7.54 d	9.02 b	8.28 C	9.70 c	9.90 c	9.80 C	9.98 e	10.45 d	10.21 C	10.89 e	10.95 e	10.92 C	11.22 d	11.10 d	11.16 C
Co-BTC	9.74 a	8.31 c	9.03 A	10.82 b	9.80 c	10.31 A	12.02 a	10.30 d	11.16 B	12.48 a	12.42 b	12.45 A	15.74 a	11.81 d	13.77 B
Cu-BTC	9.51 a	8.66 c	9.08 A	10.91 a	10.64 b	10.78 A	11.49 b	11.84 a	11.66 A	12.34 b	12.12 c	12.23 A	12.73 c	15.60 a	14.16 A
Mean	8.39 A	8.48 A		10.05 A	9.63 B		10.75 A	10.50 A		11.49 A	11.31 A		12.89 A	12.37 B	

Values followed by the same letter in columns are not different at *p* < 0.05 by Duncan’s multiple range tests

The effect of spray treatments on the weight of the *faba bean* shoot mass, which was sampled at various age stages-specifically, 3, 6, 9, 12, and 15 days following the foliar spraying procedure-is displayed in Table 3. According to the table’s statistics, the weight of the two faba bean kinds’ shoot masses increased as their ages increased. The faba bean seedlings’ green mass weight increased most effectively with the Cu-BTC spray treatment. This occurred on days 6, 9, and 15 following spraying; the increase was 1.68, 1.86, and 1.38 times greater than the control for each age stage independently. Referring to the comparison between the varieties, the Giza 716 variety outperformed the other variety in terms of the weight of the vegetative mass at every age stage at which the vegetative samples were collected, according to the data presented in the same table. In terms of the interaction between the varieties and the spray treatments, the data in the same table showed that the weight of the vegetative mass of the Sakha 4 variety rose when the Ni-BTC and Co-BTC spray treatment were applied when samples were obtained three and twelve days after the spray application, respectively. On the other hand, the weight of the vegetative mass of the Sakha 4 variety increased when the Cu-BTC spray treatment was applied when samples were collected six, nine, and fifteen days following the foliar spray application. However, the Giza 716 variety showed the best green mass weight when the Co-BTC spray treatment was applied to the *faba* bean seedlings when green samples were taken six, and nine days after the foliar spray was applied.

**Table 3:** Effect of foliar application of MOFs on fresh weight dynamics in faba bean plants over time

Foliar applications	Fresh shoot weight (g)														
	Three days			Six days			Nine days			Twelve days			Fifteen days		
	Sakha 4	Giza 716	Mean	Sakha 4	Giza 716	Mean	Sakha 4	Giza 716	Mean	Sakha 4	Giza 716	Mean	Sakha 4	Giza 716	Mean
Control	3.53 e	3.38 e	3.45 D	4.67 f	4.65 f	4.66 C	4.97 f	5.34 e	5.16 D	6.17 g	7.56 f	6.86 D	9.04 f	9.23 f	9.13 D
Ni-BTC	6.14 c	6.56 b	6.35 B	6.44 d	9.13 a	7.78 A	6.56 d	10.22 a	8.39 C	8.46 e	12.14 a	10.30 B	10.50 e	13.45 b	11.98 B
Cr-BTC	5.04 d	6.37 c	5.70 C	6.09 e	8.35 b	7.22 B	7.59 c	9.48 b	8.53 C	8.94 e	10.48 c	9.71 C	8.95 f	14.37 a	11.66 C
Co-BTC	6.01 c	6.72 b	6.37 B	6.70 d	8.93 a	7.82 A	7.39 c	10.87 a	9.13 B	10.12 d	10.58 c	10.35 B	11.31 d	12.36 c	11.84 B
Cu-BTC	5.73 d	7.37 a	6.55 A	6.85 c	8.84 b	7.84 A	9.34 b	9.87 b	9.61 A	10.00 d	11.65 b	10.83 A	11.50 d	13.65 b	12.58 A
Mean	5.29 B	6.08 A		6.15 B	7.98 A		7.17 B	9.15 A		8.74 B	10.48 A		10.26 B	12.61 A	

Values followed by the same letter in columns are not different at *p* < 0.05 by Duncan’s multiple range tests

The effect of nickel conjugated with metal organic frameworks (Ni-BTC), chromium conjugated with metal organic frameworks (Cr-BTC), cobalt conjugated with metal organic frameworks (Co-BTC), and copper conjugated with metal organic frameworks (Cu-BTC) spray treatments on the dry weight of the vegetative shoot at various age stages is displayed in Table 4. Samples were collected following the foliar spraying procedure at intervals of 3, 6, 9, 12, and 15 days. The data in the table indicate that the dry weight of the vegetative shoot rises with the advancement of the plant age. The table’s data also demonstrated that Cu-BTC was the most effective spraying treatment, improving the dry weight of the green mass when samples were collected nine, twelve, and fifteen days after the spraying procedure. This increase was estimated to be 1.57, 1.54, and 1.40 times greater than the control group for each age group, respectively. When comparing the two faba bean varieties, the Giza 716 variety outperformed the Sakha 4 variety in terms of raising the dry weight of the green mass at all different age stages in which the samples were collected. According to the data in the table and the interaction between varieties and spray treatments, the Sakha 4 variety was successful in increasing the dry weight of the vegetative shoot when the Cu-BTC spray treatment was applied when samples were collected 9, and 15 days after the spraying process. As for the Giza 716 variety, it succeeded in increasing the dry weight of the vegetative shoot when applying the Cu-BTC spray treatment, and this was in samples taken 3, 6, 12, and 15 days after applying the foliar spraying process. This study demonstrated the significance of copper in raising the fresh weight of the shoot, which is entirely in line with earlier research [22, 45], which employed Cu-BTC sprayed on *Avena sativa* seedlings to boost leaf biomass. Copper efficiently affects biological processes in plants, enhancing biomass formation and growth-promoting activity via activating a variety of enzymes, including polyphenol oxidase enzymes, and the electron transfer mechanism in photosynthesis [17]. Furthermore, because copper is involved in numerous redox reactions and the structural creation of the Fe-Cu cluster, copper is a crucial micronutrient for plants [46]. 

**Table 4:** Effect of foliar application of MOFs on dry weight dynamics in faba bean plants over time

Foliar applications	Dry shoot weight (g)														
	Three days			Six days			Nine days			Twelve days			Fifteen days		
	Sakha 4	Giza 716	Mean	Sakha 4	Giza 716	Mean	Sakha 4	Giza 716	Mean	Sakha 4	Giza 716	Mean	Sakha 4	Giza 716	Mean
Control	0.29 f	0.36 e	0.33 D	0.42 f	0.46 e	0.44 C	0.54 e	0.57 e	0.56 E	0.57 f	0.74 e	0.66 D	0.89 e	0.99 d	0.94 D
Ni-BTC	0.59 b	0.50 c	0.54 B	0.66 b	0.63 c	0.65 B	0.73 d	0.92 a	0.82 C	0.96 b	0.99 b	0.98 B	1.07 c	1.28 b	1.18 B
Cr-BTC	0.44 d	0.58 b	0.51 C	0.60 d	0.67 b	0.64 B	0.75 d	0.79 c	0.77 D	0.86 d	0.94 c	0.90 C	0.96 d	1.05 c	1.01 C
Co-BTC	0.53 c	0.57 b	0.55 B	0.61 d	0.76 a	0.68 A	0.77 c	0.94 a	0.85 B	0.97 b	1.06 a	1.02 A	1.08 c	1.32 a	1.20 B
Cu-BTC	0.50 c	0.71 a	0.61 A	0.60 d	0.78 a	0.69 A	0.88 b	0.89 b	0.88 A	0.85 d	1.19 a	1.02 A	1.27 b	1.38 a	1.32 A
Mean	0.47 B	0.55 A		0.58 B	0.66 A		0.73 B	0.82 A		0.84 B	0.99 A		1.05 B	1.20 A	

Values followed by the same letter in columns are not different at *p* < 0.05 by Duncan’s multiple range tests

When samples were collected at different phases of the foliar spraying process, the results displayed in Tables 5 and 6 demonstrate the magnitude of the impact of spraying treatments with Ni-BTC, Cr-BTC, Co-BTC, and Cu-BTC on the fresh and dry weight of the faba bean root system. The data in the same tables showed that the spraying treatment containing Cr-BTC was the most successful in increasing the fresh and dry weight of the faba bean root system when samples were taken at intervals of 9, 12, and 15 days after the spraying operation. The estimated fresh root weight increases were 1.44, 1.59, and 1.85 times, and the estimated dry root weight increases were 1.19, 1.62, and 1.93 times over the control for the age stages at which the samples were taken, which were 9, 12, and 15 days after the foliar spraying procedure. Regarding the variations between the varieties, the table’s findings demonstrated that, when samples were collected 9, 12, and 15 days after the spraying procedure, the Giza 716 variety outperformed the Sakha 4 variety in raising the root system’s fresh weight. Samples collected 12 days following the spraying procedure in the same scenario demonstrated that the Giza716 variety performed better than the other variety in terms of raising the dry weight of the root system. The dry weight of the root system did not significantly differ between the two types in the samples collected at the remaining age stages. When samples were taken after 3, 6, 12, and 15 days of the spraying procedure, the data in the same tables also showed that the Sakha 4 variety could increase the fresh weight of the root system when sprayed with the Cr-BTC treatment, showing the interaction between varieties and spray treatments. Additionally, when samples were collected after 15 days of the two external spraying procedures, the dry weight of the Sakha 4 variety’s root system increased as a result of the Cr-BTC spraying. Except for the fresh weight after three days of the spraying procedure, the Giza 716 variety was able to improve both the fresh and dried weight of the root system after administering the Ni-BTC spray treatment when samples were taken at all age stages of the spraying process. The information gathered for this study is entirely consistent with earlier research [47], adding 5 mg kg^−1^ of chromium accelerated the pace at which organic matter burned and the rate at which soil nitrified. In pot culture tests, chromium addition to the soil increased the amount of nitrogen in pea seeds and the nitrogen-fixing capacity of the inoculated plants [48]. Reducing chromium toxicity may be aided by chromium nanopreparation. Because the plant lacks a particular mechanism for chromium transport, chromium accumulates in the roots as it is absorbed by certain critical element transporters from the soil [49]. Through the disruption of gibberellic acid-related pathways and the stimulation of signaling cascades mediated by ethylene, abscisic acid, and jasmonate, chromium influences gene expression. Therefore, chromium plays a part in controlling how plant hormones function [44]. While up-regulated genes are implicated in xenobiotics, amino acid metabolism, secondary metabolite production, membrane transport, and signal transduction, down-regulated genes are associated with energy metabolism and cell growth. Chromium activated a group of genes linked to calcium, MAPKs, CDPK-like kinases, and reactive oxygen species-all of which are important components of signal transduction pathways and cognition [50]. In a similar vein, a number of miRNAs that were markedly elevated in tobacco plants under chromium stress were discovered [51].

**Table 5:** Effect of foliar application of MOFs on fresh root weight dynamics in faba bean plants over time

Foliar applications	Fresh root weight (g)														
	Three days			Six days			Nine days			Twelve days			Fifteen days		
	Sakha 4	Giza 716	Mean	Sakha 4	Giza 716	Mean	Sakha 4	Giza 716	Mean	Sakha 4	Giza 716	Mean	Sakha 4	Giza 716	Mean
Control	1.95 a	1.30 e	1.63 A	1.74 c	1.47 e	1.61 D	1.68 e	1.65 e	1.67 D	1.42 f	1.96 e	1.69 D	1.24 h	2.53 f	1.89 D
Ni-BTC	1.44 d	1.27 f	1.36 C	1.63 d	2.17 a	1.89 B	1.57 f	2.49 a	2.03 C	2.18 d	3.14 a	2.66 A	2.55 f	3.55 b	3.05 B
Cr-BTC	1.60 b	1.63 b	1.61 A	2.07 a	1.77 c	1.92 A	2.35 b	2.46 a	2.41 A	2.87 b	2.51 c	2.69 A	3.68 a	3.32 c	3.50 A
Co-BTC	1.46 d	1.59 c	1.52 B	1.68 d	1.64 d	1.66 C	2.38 b	1.94 d	2.16 B	2.72 b	2.21 d	2.47 B	3.06 e	2.37 g	2.71 C
Cu-BTC	1.36 e	1.09 g	1.23 D	1.37 f	1.94 b	1.66 C	1.84 d	2.24 c	2.04 C	2.11 d	2.57 c	2.34 C	3.04 e	3.12 d	3.08 B
Mean	1.56 A	1.37 B		1.70 A	1.79 A		1.97 B	2.16 A		2.26 B	2.48 A		2.71 B	2.98 A	

Values followed by the same letter in columns are not different at *p* < 0.05 by Duncan’s multiple range tests

**Table 6:** Effect of foliar application of MOFs on dry root weight dynamics in faba bean plants over time

Foliar applications	Dry root weight (g)														
	Three days			Six days			Nine days			Twelve days			Fifteen days		
	Sakha 4	Giza 716	Mean	Sakha 4	Giza 716	Mean	Sakha 4	Giza 716	Mean	Sakha 4	Giza 716	Mean	Sakha 4	Giza 716	Mean
Control	0.32 a	0.22 d	0.27 A	0.28 e	0.30 c	0.29 C	0.27 f	0.36 c	0.32 C	0.19 g	0.38 d	0.29 D	0.18 f	0.39 e	0.28 D
Ni-BTC	0.24 c	0.27 b	0.25 B	0.29 d	0.35 a	0.32 A	0.31 e	0.42 a	0.37 A	0.36 f	0.49 a	0.42 C	0.39 e	0.54 b	0.47 C
Cr-BTC	0.21 d	0.19 e	0.19 D	0.32 b	0.28 e	0.30 B	0.39 b	0.37 c	0.38 A	0.46 c	0.47 b	0.47 A	0.58 a	0.50 c	0.54 A
Co-BTC	0.28 b	0.22 d	0.25 B	0.33 b	0.30 c	0.31 A	0.40 a	0.33 d	0.36 B	0.47 b	0.39 d	0.43 B	0.51 c	0.41 d	0.46 C
Cu-BTC	0.25 c	0.17 f	0.21 C	0.29 d	0.26 f	0.28 C	0.38 b	0.32 d	0.35 B	0.44 c	0.38 e	0.41 C	0.58 a	0.40 d	0.49 B
Mean	0.26 A	0.21 B		0.31 A	0.30 A		0.35 A	0.36 A		0.38 B	0.42 A		0.44 A	0.45 A	

Values followed by the same letter in columns are not different at *p* < 0.05 by Duncan’s multiple range tests

The information shown in Table 7 illustrates how the size of the faba bean leaves is affected by Ni-BTC, Cr-BTC, Co-BTC, and Cu-BTC spraying treatments. The findings showed that when the age stage increased, the leaf size on the two faba bean varieties increased as well. Additionally, the data in the table indicates that the foliar spraying technique with Cu-BTC increased the leaf area by 1.67, 1.60, 1.51, 1.42, and 1.46 times, respectively, compared to the control for each age stage when samples were taken after 3, 6, 9, 12 and 15 days. The Giza 716 variety expanded the leaf size at every age stage where samples were collected, which helped to explain the variance across variations. When samples were taken 3, 6, 9, and 12 days after the application of the Cu-BTC foliar spray treatment, the findings in the same table demonstrated that the Giza 716 and Sakha 4 types were successful in expanding the leaf surface area. The purpose of this was to ascertain how spray treatments and varieties interacted. In this study, environmental elements like external spray treatments and genetic factors that promote cell expansion and cell cycle activity tightly regulate the ultimate size of plant leaves. These findings completely concur with earlier research [52], following an external copper spray, the faba bean leaves’ surface area increased, which could be because of: The presence of tiny levels of copper in the culture medium facilitates cellular metabolism. As an enzyme cofactor, this metal is involved in numerous biochemical processes and is essential for respiration, photosynthesis, ethylene sensing, and antioxidant systems [53].

**Table 7:** Effect of foliar application of MOFs on leave size dynamics in faba bean plants over time

Foliar applications	Leave size (cm^2^)														
	Three days			Six days			Nine days			Twelve days			Fifteen days		
	Sakha 4	Giza 716	Mean	Sakha 4	Giza 716	Mean	Sakha 4	Giza 716	Mean	Sakha 4	Giza 716	Mean	Sakha 4	Giza 716	Mean
Control	12.46 f	11.85 f	12.15 D	14.07 f	12.34 g	13.20 D	15.85 e	13.04 f	14.44 D	17.44 f	15.05 g	16.24 D	18.83 e	17.17 f	18.00 D
Ni-BTC	14.65 e	18.17 b	16.41 C	17.42 d	21.05 b	19.24 B	18.05 d	22.88 b	20.46 B	19.51 d	23.15 b	21.33 B	25.74 c	24.88 c	25.31 B
Cr-BTC	16.82 c	15.96 c	16.39 C	15.76 e	19.22 c	17.49 C	16.47 e	19.92 c	18.20 C	17.56 f	22.65 b	20.11 C	18.07 e	24.47 c	21.27 C
Co-BTC	15.25 d	18.65 b	16.95 B	16.20 e	22.97 a	19.59 B	17.07 d	24.14 a	20.61 B	18.07 e	25.23 a	21.65 B	21.45 d	29.48 a	25.46 B
Cu-BTC	17.92 b	22.74 a	20.33 A	19.18 c	23.06 a	21.12 A	19.82 c	23.86 a	21.84 A	21.86 c	24.14 a	23.00 A	26.47 b	26.17 b	26.32 A
Mean	15.42 B	17.47 A		16.53 B	19.73 A		17.45 B	20.77 A		18.89 B	22.04 A		22.11 B	24.44 A	

Values followed by the same letter in columns are not different at *p* < 0.05 by Duncan’s multiple range tests

Following sample collection at various foliar spraying age stages, Table 8 illustrate the effect of Ni-BTC, Cr-BTC, Co-BTC, and Cu-BTC spraying treatments on the diameter of the faba bean’s stem. The results in the table showed that at every step of the vegetative sample collecting procedure, the Cu and Co treatments worked better than the others in terms of increasing the stem diameter. The stem diameter increased by 1.41 and 1.31 following Cu and Co spraying, respectively, particularly when samples were taken six days after the spraying. Across the different age stages when samples were taken after the foliar spraying technique, the Giza 716 variety outperformed the Sakha 4 variety in terms of developing stem diameter. The results demonstrated that the Sakha 4 and Giza 716 varieties were successful in increasing stem diameter when the Cu-BTC spray treatment was applied during the various age stages of plant sample collection except the Giza 716 variety at 12 days after spraying, taking into account the interaction between spray treatments and varieties. The findings of this study are in line with earlier research [54], they reported that copper contributes to the movement of nutrients and water, which helps the plant stem’s diameter to increase. Cobalt functions as a dual factor that, depending on the dose given to the plant, may either promote or inhibit plant growth [55, 56]. The following factors could be responsible for the growth-stimulating effect following very low doses of cobalt spray treatment: Cobalt’s interactions with iron, nickel, and zinc are crucial for preserving cellular homeostasis and plant development is encouraged [10]. Additionally, applying cobalt to plants can improve their ability to withstand stress and raise their water content, which will boost agricultural productivity and growth [57, 58]. Likewise, cbalt probably affects the processes of cell division and elongation, which leads to larger stems [59, 60]. Also, applying cobalt to leaves increases their relative water content and decreases electrolyte leakage, which suggests better water status and membrane integrity. This results in larger cells, which in turn increases the diameter of the plant stem [61].

**Table 8:** Effect of foliar application of MOFs on stem size dynamics in Faba bean plants over time

Foliar applications	Stem size (cm^2^)														
	Three days			Six days			Nine days			Twelve days			Fifteen days		
	Sakha 4	Giza 716	Mean	Sakha 4	Giza 716	Mean	Sakha 4	Giza 716	Mean	Sakha 4	Giza 716	Mean	Sakha 4	Giza 716	Mean
Control	1.63 e	1.47 f	1.55 D	1.77 f	1.67 g	1.72 D	1.80 f	1.79 f	1.79 C	1.88 f	1.87 f	1.88 C	1.95 f	2.06 f	2.00 D
Ni-BTC	2.03 c	2.10 b	2.07 B	2.03 d	2.37 b	2.20 B	2.19 e	2.46 a	2.33 B	2.34 d	2.56 b	2.45 B	2.59 c	2.58 c	2.58 B
Cr-BTC	1.66 e	2.21 b	1.94 C	1.97 e	2.30 c	2.13 C	2.24 d	2.40 b	2.32 B	2.31 e	2.59 b	2.45 B	2.34 e	2.63 b	2.48 C
Co-BTC	1.96 d	2.25 a	2.11 A	2.17 d	2.32 c	2.25 B	2.28 d	2.40 b	2.34 B	2.38 d	2.60 a	2.49 A	2.56 d	2.66 a	2.61 A
Cu-BTC	2.02 c	2.23 a	2.13 A	2.45 a	2.39 b	2.42 A	2.38 c	2.48 a	2.43 A	2.47 c	2.51 c	2.49 A	2.59 c	2.68 a	2.63 A
Mean	1.86 B	2.05 A		2.08 B	2.21 A		2.18 B	2.31 A		2.28 B	2.43 A		2.41 B	2.52 A	

Values followed by the same letter in columns are not different at *p* < 0.05 by Duncan’s multiple range tests

**Fig. 6:**
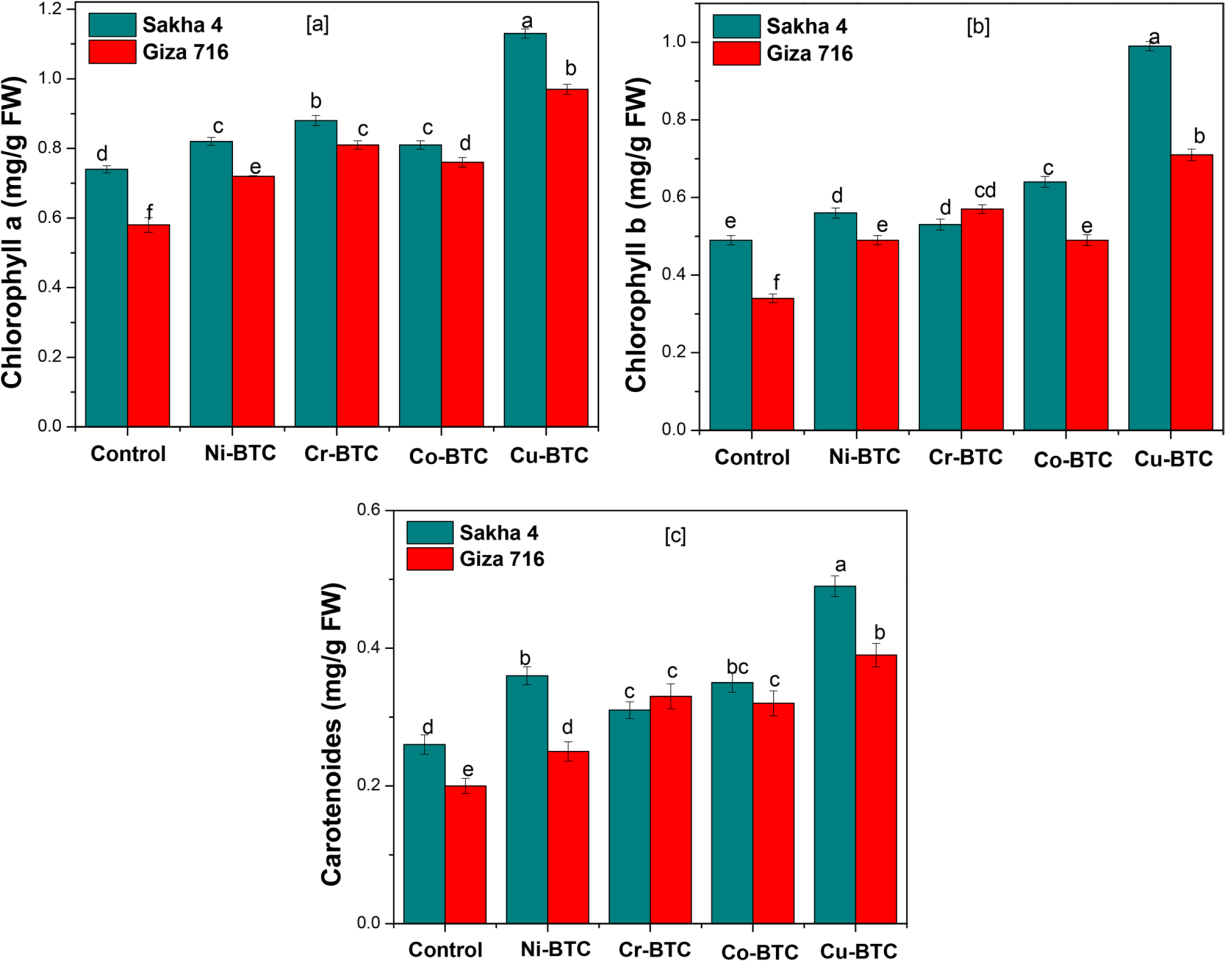
Effect of external spraying with metal-benzene tricarboxylate (M-BTC) on photosynthetic pigment content in faba bean leaves (**a**) chlorophyll a, (**b**) chlorophyll b and (**c**) carotenoids, 15 days after spraying. Data are from three replicate experiments. Error bars are standard deviations (SD) with *n* = 3. * *p* value < 0.05

The information in Table 9 and Fig. 6 show how the Ni-BTC, Cr-BTC, Co-BTC, and Cu-BTC spray treatments affected the quantity of photosynthetic pigments, particularly chlorophyll a, b, and carotenoids, in fresh leaves after 15 days of foliar spraying. The results demonstrated that the Cu-BTC spray treatment outperformed the other spray treatments in raising the amounts of carotenoids and chlorophyll a and b in faba bean leaves by 1.91, 1.59, and 2.07 times, respectively, in comparison to the control group. When Cr-BTC was sprayed, the amounts of photosynthetic pigments chlorophyll a, b, and carotenoids rose by 1.27, 1.34, and 1.39, respectively. Concurrently, the levels of carotenoids, chlorophyll b, and chlorophyll a in the leaves increased marginally by 1.43, 1.37 and 1.18 times, 1.35, 1.27 and 1.17 times, when Co-BTC and Ni-BTC were sprayed, respectively, in comparison to unsprayed plants. The Sakha 4 variety outperformed the Giza 716 variety in terms of increasing the amounts of carotenoids and chlorophyll a and b in fresh leaves. Regarding the interplay between the varieties and the spray treatments, the same table’s results demonstrated that the faba bean varieties Sakha 4 and Giza 716 were superior in raising the levels of carotenoids, chlorophyll a, and b when the Cu-BTC spray treatment was used. Cu is essential for the electron transport mediated by plant photosystem II (PSII), which is involved in the photolysis of water molecules in photosynthetic cells [13]. In their earlier research on radish and canola plants, respectively, cobalt spray treatment increased the amount of chlorophyll a, b, and total chlorophyll and carotenoides in the leaves. Photosynthetic efficiency was increased with external cobalt spraying [55, 62]. In order to retain the best possible photosynthetic performance, cobalt may also promote the synthesis of chlorophyll and shield its molecules from deterioration [62, 63]. Furthermore, Many proteins and enzymes involved in the electron transport chain, oxygenation processes, and charge accumulation use copper as a structural element and catalyst to promote the completion of photosynthesis. Copper increases the rates of photosynthesis by catalyzing the creation of cytochrome oxidase and plastocyanin. Copper is required for PS2 activity in order to maintain the correct quantity and composition of pigments and peptides in PS2, as well as the quantity and degree of fatty acid saturation in the thylakoids and PS2 complex, and to serve as a structural component of the active PS2 complex [64].

**Table 9:** Effect of foliar application of MOFs on photosynthetic pigments (mg/g FW) in Faba bean leaves after 15 days from treatments

Foliar applications	Photosynthetic pigments (mg/g FW)								
	Chlorophyll (a)			Chlorophyll (b)			Carotenoids		
	Sakha 4	Giza 716	Mean	Sakha 4	Giza 716	Mean	Sakha 4	Giza 716	Mean
Control	0.74 d	0.58 f	0.66 D	0.49 e	0.34 f	0.41 D	0.26 d	0.20 e	0.23 D
Ni-BTC	0.82 c	0.72 e	0.77 C	0.56 d	0.49 e	0.52 C	0.36 b	0.25 d	0.31 C
Cr-BTC	0.88 b	0.81 c	0.84 B	0.53 d	0.57 cd	0.55 B	0.31 c	0.33 c	0.32 C
Co-BTC	0.81 c	0.76 d	0.78 C	0.64 c	0.49 e	0.56 B	0.35 bc	0.32 c	0.33 B
Cu-BTC	1.13 a	0.97 b	1.05 A	0.99 a	0.71 b	0.85 A	0.49 a	0.39 b	0.44 A
Mean	0.88 A	0.77 B		0.64 A	0.52 B		0.35 A	0.29 B	

Values followed by the same letter in columns are not different at *p* < 0.05 by Duncan’s multiple range tests

**Fig. 7:**
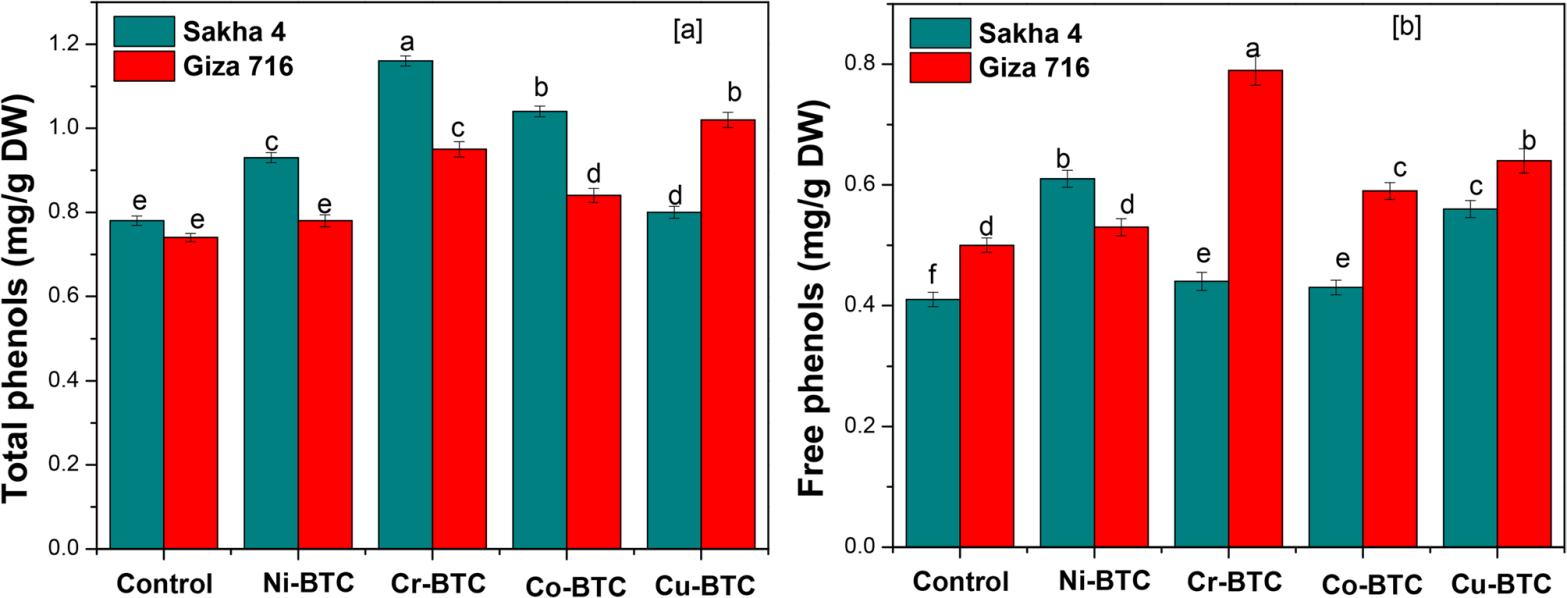
Effect of external spraying with metal-benzene tricarboxylate (M-BTC) on the phenolic content of faba bean leaves (**a**) total phenols, (**b**) free phenols, 15 days after spraying. Data are from three replicate experiments. Error bars are standard deviations (SD) with *n* = 3. * *p* value < 0.05

Table 10 and Fig. 7 show the amount of total and free phenols in the dried leaves faba bean after 15 days of external spray treatments with Ni-BTC, Cr-BTC, Co-BTC, and Cu-BTC. When the spray treatment with Cr-BTC was applied, the level of total and free phenols in the dry leaves increased 1.38 times more than the control, according to the data. Following Co-BTC, Cu-BTC, and Ni-BTC spraying, the dry leaf content of total phenols increased by 1.24, 1.20, and 1.13 times, respectively, in comparison to the control. Furthermore, when faba bean leaves were sprayed with Cu-BTC, Ni-BTC, and Co-BTC, their free phenol content increased by 1.33, 1.27, and 1.13 times, respectively, in comparison to the control. The results showed that while variety Giza 716 did better at raising the free phenol content, variety Sakha 4 was successful in increasing the total phenols, taking into account the competition between varieties to raise the dry leaf phenol content. The results in the table shed insight into the relationship between varieties and spray treatments, showing that when the spray treatment with Cr-BTC is applied, the total phenols in the leaves of variety Sakha 4 increase. Similarly, the amount of total phenols increased when Cu-BTC was sprayed on the Giza 716 variety’s leaves. When Ni-BTC was sprayed on the Sakha 4 variety’s leaves, the amount of free phenols likewise rose. However, when the Giza 716 variety was sprayed with Cr-BTC, the amount of free phenols rose in comparison to the control for both. Copper and nickel spray treatments increased the total phenolic content, with nettle and basil plants the largest increases, are consistent with the previously obtained results [9, 65]. Plants naturally contain substances called polyphenols, which contribute to the plants’ inherent antioxidant capacity [66]. Additionally, the correlation between *Solidaga canadensis*’s phenolic component content and the concentration of specific nutrients was investigated [67]. When nickel was applied, marigold’s antioxidant activity increased noticeably. When nickel was added to the growth media, *Matricaria chamomilla* accumulated phenolic acids, including the crucial antioxidant chlorogenic acid, around four times as much. As a result, it has been proposed that nickel might possess phenolic compound antioxidant qualities [68, 69]. Plants may have higher levels of phenolic compounds as a result of heavy metals penetrating their tissues and producing more reactive oxygen species (ROS), which in turn triggers the plant defense system and increases the synthesis of non-enzymatic antioxidants such as phenols [70, 71]. Nettle and basil plants had higher levels of phenolic compounds overall when copper was added to the soil [65].

**Table 10:** Effect of foliar application of MOFs on phenolic content (mg/g DW) in leaves of faba bean plants after 15 days from treatments

Foliar applications	Phenolic content (mg/g DW)					
	Total phenolic			Free phenolic		
	Sakha 4	Giza 716	Mean	Sakha 4	Giza 716	Mean
Control	0.78 e	0.74 e	0.76 D	0.41 f	0.50 d	0.45 C
Ni-BTC	0.93 c	0.78 e	0.86 C	0.61 b	0.53 d	0.57 B
Cr-BTC	1.16 a	0.95 c	1.05 A	0.44 e	0.79 a	0.62 A
Co-BTC	1.04 b	0.84 d	0.94 B	0.43 e	0.59 c	0.51 B
Cu-BTC	0.80 d	1.02 b	0.91 B	0.56 c	0.64 b	0.60 A
Mean	0.94 A	0.87 B		0.45 B	0.61 A	

Values followed by the same letter in columns are not different at *p* < 0.05 by Duncan’s multiple range tests

**Fig. 8:**
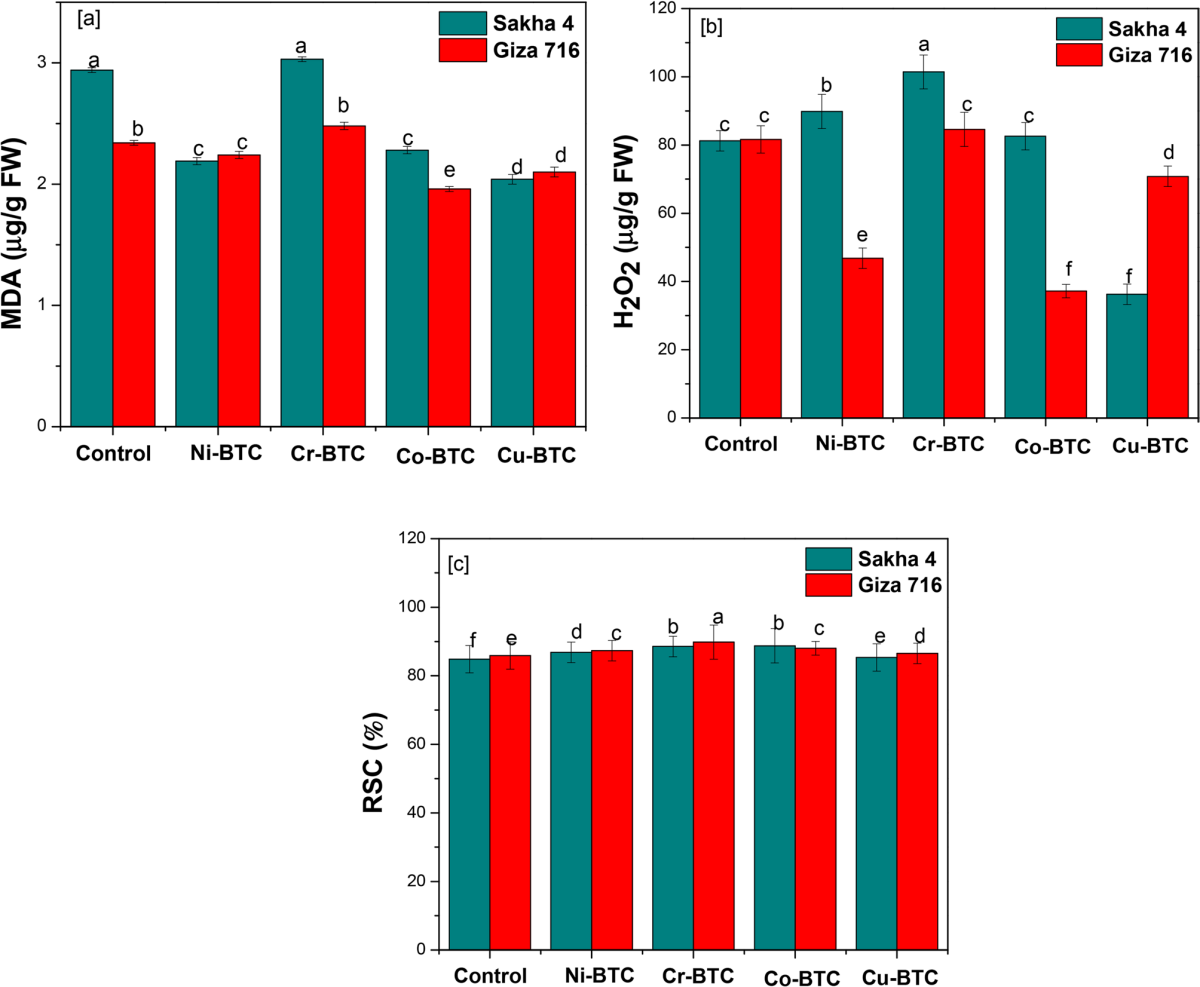
Effect of external spraying with metal-benzenetricarboxylates (M-BTCs) on physiological stress indices in fresh leaves of faba bean (**a**) Malondialdehyde, [**b**] Hydrogen peroxide, (**C**) Radical scavenging capacity percentage, 15 days after spraying. Data are from three replicate experiments. Error bars are standard deviations (SD) with *n* = 3. * *p* value < 0.05

Table 11 and Fig. 8 display the values of physiological stress indices MDA, H_2_O_2_, and RSC% in the leaves of a faba bean under the influence of spraying treatments with Ni-BTC, Cr-BTC, Co-BTC, and Cu-BTC. The results showed that the Cu-BTC spraying treatment was effective in reducing the percentages of MDA and H_2_O_2_ by 21.59% and 34.26%, respectively, compared to the control. Additionally, spraying treatments with Co-BTC and Ni-BTC decreased MDA rates by 19.70%, and 16.29%, respectively. Additionally, as compared to the control, the same treatments decreased the H_2_O_2_ concentration of fresh leaves by 26.46%, and 16.09%, respectively. On the contrary, the Cr spraying caused the rate of MDA and H_2_O_2_ to rise by 4.54% and 14.21% greater than the control, respectively. The Cr-BTC spraying treatment raised the free radical scavenging rate by 89.17%, followed by the Co-BTC treatment (88.38%), Ni-BTC, and Cu-BTC (87.08%), and then the (85.94%), according to the results in the same table respectively. In terms of variety variation, the same table’s results revealed no discernible variations between Sakha 4 and Giza 716 in terms of the value of MDA and percentage of RSC in the leaves, but the Giza 716 variety was able to lower the value of H_2_O_2_ in comparison to the other variety. Taking into consideration the interaction between varieties and spray treatments, the results in the same table showed that the Sakha 4 variety was able to achieve the highest rates of lowering MDA, H_2_O_2_ and RSC% when the Cu-BTC spray treatment was applied to the seedlings. Meanwhile, the Giza 716 variety was able to lower the rates of MDA and H_2_O_2_ while increasing the percentage of RSC when the Co-BTC spray treatment was used. The aforementioned findings are entirely consistent with earlier research [72], which indicates that reactive oxygen species (ROS) elimination may involve copper ions. By reducing the generation and accumulation of free radicals through its involvement in boosting the activity of antioxidant enzymes such as Cu/Zn-SOD [73], ethylene receptor, laccase, polyphenol oxidase, and other multiple copper oxidases [74], copper helps plants become more resilient and tolerant to stress. Copper also encourages the oxidation of putrescine, which produces hydrogen peroxide (H_2_O_2_), which is required for cell wall strengthening, lignification, protein cross-linking [75]. Additionally, copper can create stable compounds that aggressively detoxify histidine and proline by forming functional groups like amino, carboxyl, and hydroxyl [76]. Furthermore, cobalt scavenges reactive oxygen species and increases the activity of antioxidant enzymes, shielding plant cells from oxidative damage and preserving cell integrity [77]. The way that glutathione, ascorbate, glutathione reeducates, and related substances work to control oxidative stress, maintain cellular homeostasis, and shield cells from oxidative damage may be impacted by cobalt [78–80]. The way that glutathione, ascorbate, glutathione reductase. Cobalt may improve overall plant health by promoting osmotic accumulation, increasing protein synthesis, and modifying the antioxidant defense system [81].

**Table 11:** Effect of foliar application of MOFs on malondialdehyde, hydrogen peroxide and radical scavenging capacity in leaves of faba bean plants after 15 days from treatments

Foliar applications	Stress physiological indices								
	MDA (µg/g FW)			H_2_O_2_ (µg/g FW)			RSC %		
	Sakha 4	Giza 716	Mean	Sakha 4	Giza 716	Mean	Sakha 4	Giza 716	Mean
Control	2.94 a	2.34 b	2.64 B	81.24 c	81.62 c	81.43 B	84.85 f	85.91 e	85.38 D
Ni-BTC	2.19 c	2.24 c	2.21 C	89.84 b	46.82 e	68.33 C	86.84 d	87.33 c	87.08 C
Cr-BTC	3.03 a	2.48 b	2.76 A	101.42 a	84.58 c	93.00 A	88.54 b	89.81 a	89.17 A
Co-BTC	2.28 c	1.96 e	2.12 D	82.57 c	37.18 f	59.88 D	88.73 b	88.02 c	88.38 B
Cu-BTC	2.04 d	2.10 d	2.07 E	36.26 f	70.80 d	53.53 E	85.34 e	86.54 d	85.94 D
Mean	2.49 A	2.22 A		78.26 A	64.20 B		86.86 A	87.52 A	

Values followed by the same letter in columns are not different at *p* < 0.05 by Duncan’s multiple range tests

The mode of MOFs action on plant growth has several mechanisms [82–84], primarily by facilitating the controlled release of nutrients and agrochemicals, improving stress resilience, and acting as antimicrobial agents. MOFs can minimize environmental impact by decreasing the amount of heavy metals used. Moreover, the metal center in MOFs plays a role in chlorophyll synthesis and other enzymatic activities related to photosynthesis. By ensuring adequate metal availability through MOFs, plants may be able to photosynthesize more efficiently, leading to increased biomass production. Cu-BTC has shown promising results in plant growth due to its unique properties such as presence of Cu as micronutrient, different morphology, and the release rate of Cu and organic linker that able to act as a nano-fertilizer. Moreover, Cu-BTC has high surface area leading to control release of Cu nutrients that can enhance nutrient uptake by plants, leading to improved growth and yield. Additionally, Cu-BTC’s potential antimicrobial properties can help protect plants from diseases, further contributing to better growth. MOFs are environmentally safe because they are biocompatible and biodegradable due to the organic linkers in their backbone, less toxic and more abundant metal ions in their nodes, and less toxic and more sustainable solvents in their synthetic protocol [85].

## Conclusion

Analogous metal-benzenetricarboxylates (M-BTCs), composed of Ni^2+^, Cr^3+^, Co^2+^, and Cu^2+^ as central metal ions, were prepared using rapid techniques with high yield. Ni-BTC, Cr-BTC, Co-BTC, and Cu-BTC were sprayed on bean plants to ensure precise process control and yield prediction in plant. Plant growth parameters such as shoot and root length, along with weight, are key indicators of plant health. Cu-BTC showed a significant treatment effect compared to the control; shoot length in Cu-BTC was 1.14 times higher than the control, while root length was 1.44 times higher. Fresh and dry shoot weights increased by 1.48 and 1.39 times, respectively. Fresh root weight, dry root weight, and leaf size increased by 1.23, 1.03, and 1.52 times, respectively, compared to the control. The results demonstrate that Cu-BTC can be used at low concentrations to increase the fresh and dry weights of the root system. Additionally, copper is essential for plant growth because it enhances vegetative development, boosts chlorophyll levels, and reduces free radical buildup in plant leaves. This study emphasizes that, besides porosity and pore structure, the central metal ions of MOFs should be carefully considered before applying these materials to various purposes, including agriculture.

## Supplementary Information

Supplementary Material 1.

## Data Availability

I do not have any research data outside the submitted manuscript file.
