# Supplementary material for: Influence of different metal-organic frameworks on agronomic traits of faba bean plants
Source: BMC Plant Biol. 2025 Oct 31;25:1485. doi: 10.1186/s12870-025-07478-7 (PMC12577044; doi:10.1186/s12870-025-07478-7)
Supplement: Supplementary file 1 — Supplementary Material 1. [file 12870_2025_7478_MOESM1_ESM.docx]

Table S1. Chemical analysis of the experimental soil and irrigation water

| a) Physical analysis of the experimental soil | | | | | | | | | | | | | | |
| --- | --- | --- | --- | --- | --- | --- | --- | --- | --- | --- | --- | --- | --- | --- |
| Particle size distribution % | | | | | | | | | | | | | | |
| Very coarse sand | Coarse sand | Medium sand | | Fine sand | Very fine sand | | Silt | Clay | | Soil texture | | | CaCO_3_ % | |
| 14.96 | 11.02 | 9.87 | | 19.09 | 36.57 | | 3.39 | 5.10 | | Sandy soil | | | 3.41 | |
| b) Chemical analysis of the experimental soil | | | | | | | | | | | | | | |
| EC dS m^-1^ | pH | | Cations (meq L^-1^) | | | | | | Anions (meq L^-1^) | | | | | |
|  |  |  | Ca ^++^ | Mg ^++^ | Na ^+^ | K ^+^ | | | CO_3_ ^--^ | | HCO_3_ ^-^ | Cl ^-^ | | SO_4_ ^--^ |
| 3.50 | 7.62 | | 3.56 | 0.67 | 30.17 | 0.98 | | | Nil | | 2.40 | 30.04 | | 2.56 |
| c) Chemical analysis of irrigation water | | | | | | | | | | | | | | |
| EC dS m^-1^ | pH | | Cations (meq L^-1^) | | | | | | Anions (meq L^-1^) | | | | | |
|  |  |  | Ca ^++^ | Mg ^++^ | Na ^+^ | K ^+^ | | | CO_3_ ^--^ | | HCO_3_ ^-^ | Cl ^-^ | | SO_4_ ^--^ |
| 0.43 | 7.84 | | 2.25 | 0.35 | 1.45 | 0.80 | | | Nil | | 1.87 | 1.44 | | 0.86 |
